# Useful Extend-release Chitosan Tablets with High Antioxidant Activity

**DOI:** 10.3390/pharmaceutics2020245

**Published:** 2010-05-27

**Authors:** Taira Yasufuku, Makoto Anraku, Yuko Kondo, Toshiyuki Hata, Junzo Hirose, Nobuyuki Kobayashi, Hisao Tomida

**Affiliations:** 1 Faculty of Pharmacy and Pharmaceutical Sciences, Fukuyama University, 1 Sanzo, Gakuen-cho, Fukuyama 729-0292, Japan; 2 Dainichiseika Color & Chemicals Mfg. Co., Ltd., 7-6, Nihonbashi Bakuro-cho 1-chome, Chuo-ku, Tokyo, 103-8383, Japan

**Keywords:** chitosan, antioxidant, molecular weight, matrix tablet, extend-release, radicals

## Abstract

The antioxidant properties of different low molecular weight (LMW) chitosans (CS1; 22 kDa, CS2; 38 kDa, CS3; 52 kDa, CS4; 81 kDa) were examined for possible use in extended-release tablets. The criteria used were the ability of the chitosans to reduce Cu^2+^, and hydroxyl and superoxide radicals and N-centered radicals derived from 1,1'-diphenyl-2-picrylhydrazyl, via the use of ESR spectrometry. CS2 showed the highest scavenging activity. CS1 and CS3, however, were much less effective and CS4 was not a viable antioxidant. The results suggest that CS2 could be useful in combating the development of oxidative stress. A series of chitosan tablets were prepared using a spray drying method and evaluated as an extended-release matrix tablet using theophylline (TPH) as a model drug. The release of TPH from the different MW chitosan tablets increased with increasing MW of the chitosan used. CS2, CS3 and CS4 showed a reasonable release activity, but CS1 showed the shortest release activity. Moreover, the CS2-TPH tablet showed the highest scavenging activity of the three chitosan tablets (CS2-CS4) using 2,2’-azinobis (3-ethylbenzothiazoline-6-sulfonic acid) radicals. These results suggest that a CS2-TPH tablet could be potentially useful in an extended-release matrix tablet with a high antioxidant activity.

## 1. Introduction

When a drug is freely soluble in water, the judicious selection of release-retarding excipients is necessary to achieve a constant *in vivo* input rate. One of the most commonly used methods for modulating drug release is to include it in a matrix system. Hydrophilic gel forming polymer matrix systems are widely used in oral controlled drug delivery to obtain a desirable drug release profile, for cost effectiveness and broad regulatory acceptance [1,2]. Indeed, hydrogels derived from natural polymers, especially polysaccharides, have been widely used because of their unique advantages such as their nontoxic, biocompatible, biodegradable properties and the fact that they are readily available [3].

Chitosan is a naturally occurring cationic copolymer comprised of glucosamine, and can be produced by the deacetylation of chitin, which is the second most abundant polysaccharide after cellulose in the world. It is widely used in a variety of pharmaceutical formulations as sustained release carrier systems such as beads, gels, films, sponges and microspheres, because of many unique properties, which include low toxicity, biocompatibility, biodegradability and mucoadhesive properties [4,5,6,7,8]. In addition, a property of particular interest for this study is the antioxidant properties of chitosan [9,10]. In a previous study, we reported that the scavenging of hydroxyl radicals by low molecular weight (LMW) chitosan inhibits the peroxidation of human serum albumin (HSA) [11]. Santhosh *et al.* [12] reported that the administration of chitosan to rats that had been treated with isoniazid or rifampicin prevented hepatotoxic lipid oxidation. Similarly, it was reported that the injection of chitosan inhibits glycerol-induced renal oxidative damage in rats by Yoon *et al*. in, 2008 [13]. In a previous study, we also showed that the administration of LMW chitosan to human volunteers prevented the oxidation of HSA *in vivo* (Anraku *et al*., 2009) [14]. Therefore, given the likelihood that LMW chitosans have antioxidative properties, they would be potentially useful as components of extended-release systems for use in drug delivery.

The aim of this study was to evaluate the antioxidant and free radical-scavenging properties of several LMW chitosan preparations in *in vitro* studies. We also evaluated the antioxidant properties of an extended-release tablet that contained combinations of LMW chitosans.

## 2. Materials and Methods

### 2.1. Materials

**Figure 1:**
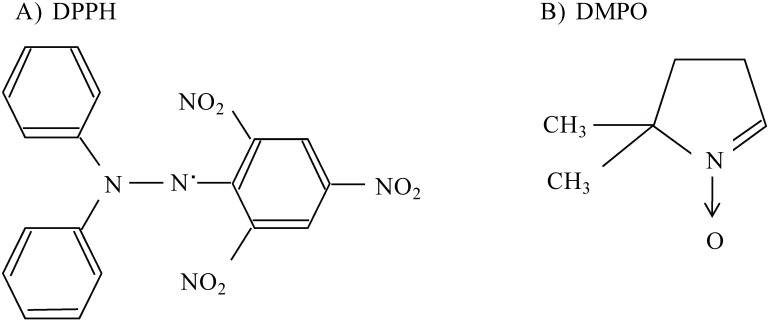
The chemical structures of DPPH and DMPO.

**Figure 2:**
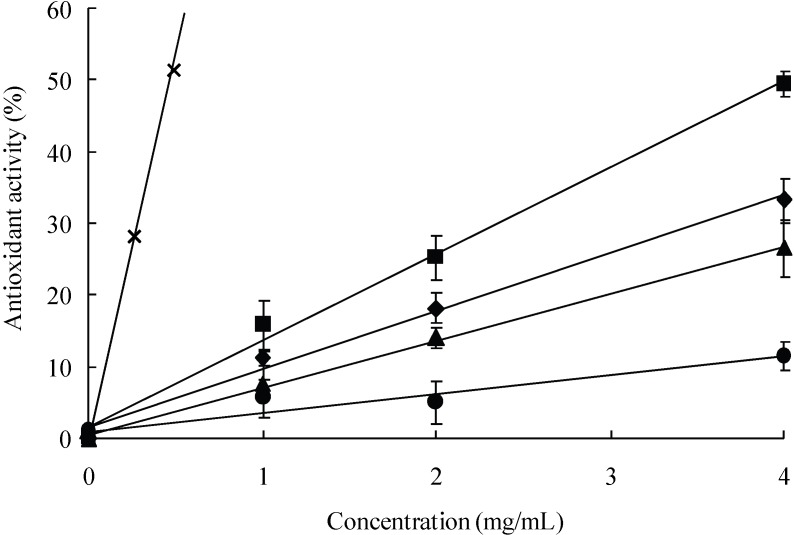
Relative effectiveness of different concentrations of the antioxidants in reducing DPPH radicals. The activities are shown relative to fully reduced DPPH (100%). The DPPH radical concentration was measured at 517 nm. CS1 (♦), CS2 (■), CS3 (▲), CS4 (●) and VC (**×**).

Low molecular weight (LMW) chitosans (CS1; 22 kDa, ; CS2; 38 kDa, ; CS3; 52 kDa, ; CS4; 81 kDa, ; deacetylation degree 90%) were generously supplied by Dainichiseika Color & Chemicals Mfg. Co., Ltd (Tokyo, Japan). Theophylline anhydrous (TPH) was purchased from Wako (Tokyo, Japan). The 1,1'-diphenyl-2-picrylhydrazyl (DPPH) (Figure 1A), 2,2’-azinobis (3-ethylbenzothiazoline-6-sulfonic acid) (ABTS), ascorbic acid (VC) and 5,5-Dimethyl-1-pyrroline N-oxide (DMPO) (Figure 1B) was supplied by Nacalai Tesque (Kyoto, Japan). All other chemicals were of the highest grade commercially available, and all solutions were prepared using deionized, distilled water. 

### 2.2. Scavenging activity of LMW chitosans on DPPH radicals

The radical scavenging activities of different concentrations of LMW chitosans were tested in ethanolic solution (10 mL of ethanol, 10 mL of 50 mM 2-(N-morpholino) ethanesulfonic acid (MES) buffer (pH 5.5) and 5 mL of 0.5 mM DPPH. Radical scavenging was estimated based on the decrease in absorbance of DPPH radicals at 517 nm [15].

### 2.3. Reducing power by using the TPAC test

The antioxidant power of the tested LMW chitosans was determined using the ‘TPAC’ test (Cosmo Bio Co., Ltd., Tokyo, Japan). In this assay, the levels of Cu^+^ produced by the reduction of Cu^++^ by the action of antioxidants present in the sample are determined. The stable complex between Cu^+^ and bathocuproine was assayed at 490 nm, with a sensitivity of 22 μmol L^-1^ of reducing power. The assay was found to be linear in the range from 1-2000 μmol L^ -1^ of uric acid (r = 0.99, *p* < 0.01). Both within-run and between-run assay variability, tested by repeatedly assaying five samples, was consistently lower than 5%.

### 2.4. ESR spectroscopy

Different radicals were generated according to previously reported procedures and spin adducts were recorded using a JES-FA electron spin resonance (ESR) spectrometer (JEOL, Tokyo, Japan) at 25 ºC. The instrument settings were as follows: magnetic field 336 ± 5 mT; sweep time 30 s; sweep width 10 mT; modulation width 0.1 mT and modulation frequency 100 kHz. Radical scavenging activity of LMW chitosans were calculated as a scavenging percentage by S = (ho−h1)/ho×100%; where, h1 and ho were ESR signal intensities in the presence and absence of test compound, respectively.

### 2.5. Hydroxyl radical assay

Fenton reaction was performed by reacting 50 μL of 10 mM FeSO_4_ and 50 μL of 10 mM H_2_O_2_ to generate ^•^OH radicals [16]. The generated radicals were trapped in 50 μL of 0.3 M DMPO in the presence of the test compound (50 μL) or the same volume of phosphate buffer (pH 7.4), which served as a control. After 2.5 min, the reaction mixture was transferred to a sealed capillary tube and the DMPO/^•^OH spin adduct was recorded at 1 mW microwave power and an amplitude of 400.

### 2.6. Superoxide radical assay

Superoxide anion radicals were generated using a UV irradiated riboflavin/EDTA system [17]. Reaction mixtures containing 0.8 mM riboflavin, 1.6 mM EDTA, 0.8 M DMPO and different concentrations of sample solutions were irradiated for 1 min under a UV lamp at 365 nm. The reaction mixture was then transferred to the cavity of the ESR spectrometer using a sealed capillary tube and the DMPO/O2•‾ spin adduct was recorded at a microwave power of 10 mW and an amplitude of 1000.

### 2.7. Preparation of extended-release matrix tablet

LMW chitosans were dissolved in a 1% aqueous acetic acid solution. The solutions were then spray dried under the following conditions: inlet temperature of 140 ºC, drying air-flow of 0.50 m^3^/min, atomizing air pressure of 50 kPa, and an outlet temperature of 90–95 ºC. The matrix particles were prepared by spray drying using a SD-1000 instrument (Tokyo Rikakikai Co., Ltd., Japan). The extended-release matrix tablets, with a total weight of 400 mg, were prepared using a mixture of theophylline and an excipient at a weight ratio of 1:1. The mixture was compressed using a hydraulic press with a 13-mm diameter. The compression force was 10 kN / cm^2^ with a dwell time of 180 s. The Spray-dried LMW chitosans were used as the excipients.

### 2.8. Dissolution of TPH from tablets and scavenging activity of chitosans-TPH tablets on ABTS radicals

Dissolution tests were carried out using a dissolution tester (DST 810, Toyama, Inc., Japan). The rate of dissolution of TPH was measured using the USP paddle method at 100 rpm using 900 mL of distilled water at 37 ºC including ABTS radical solutions. Stable ABTS cation radicals (ABTS^•+^) were generated by oxidation with potassium persulfate. The reaction mixture contained 70 mM potassium persulfate and 2 mM ABTS in 900 mL of distilled water. The stable ABTS^•^^+^ radical was generated on standing for 24 h and was used in the assay. The reaction of any radicals present with the ABTS^•+^ was estimated from the decrease in its absorbance at 734 nm [18]. At the same time, the concentration of TPH was determined using a UV spectrometer (Shimadzu Scientific Instrument, Kyoto, Japan) at a wavelength of 271 nm.

### 2.9. Statistics

Statistical significance was evaluated by ANOVA followed by the Newman-Keuls test for comparison among >2 mean values. For all analyses, values of *p* < 0.05 were regarded as statistically significant. Results are reported as the mean ± SEM.

## 3. Results and Discussion

### 3.1. Scavenging activity of LMW chitosans on DPPH radicals

As shown in Figure 2, the scavenging activity of several of the LMW chitosans on DPPH radicals is substantial and concentration-dependent. The antioxidative effect was observed to be in the order: CS2 > CS1 > CS3 >> CS4 (mg/mL). CS2 showed a particularly high antioxidative effect but did not reach the level of VC. Moreover, as shown in Table 1, the IC_50_ values for the LMW chitosans were 4.02, 6.00, 7.54, 18.73 mg/mL, respectively. Collectively, these results demonstrate that CS2 has the ability to scavenge oxygen- and nitrogen-centered radicals and suggest that its antioxidant potential, as has been shown in other systems, may be due, at least in part, to this property.

**Table 1:** Scavenging of DPPH radicals by different LMW chitosans.

Antioxidant	DPPH
	IC_50 _(mg/mL)^ a^
CS1	6
CS2	4.02
CS3	7.54
CS4	>10

^a^ Relative radical trapping ability was calculated using 0.5mM DPPH.

### 3.2. Reducing power of LMW chitosans by using the TPAC test

**Figure 3:**
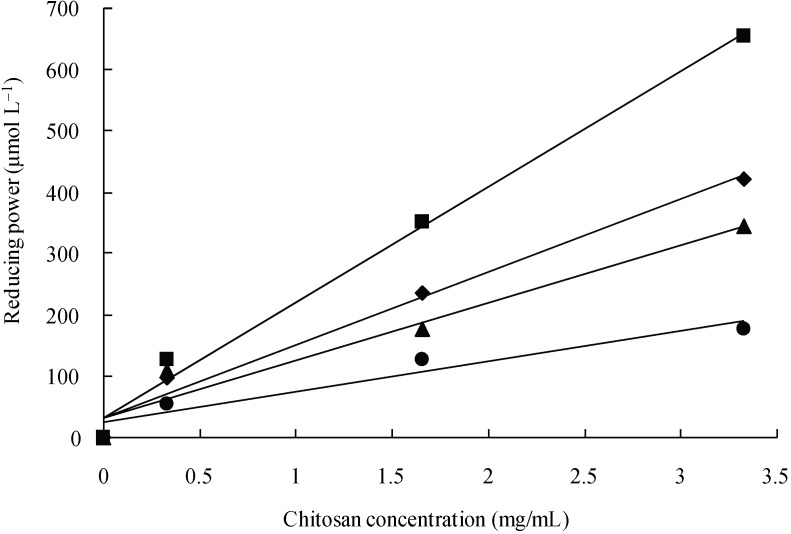
Reducing power of different LMW chitosans in the TPAC test. CS1 (♦), CS2 (■), CS3 (▲), and CS4 (●).

Figure 3 shows data on the reducing power of the LMW chitosans. The reducing power was observed to be in the order: CS2 > CS1 > CS3 > CS4 (μmol/L). These results suggest that CS2 is a good reducing agent. 

### 3.3. Radical scavenging potency of LMW chitosans by ESR spectroscopy

**Figure 4:**
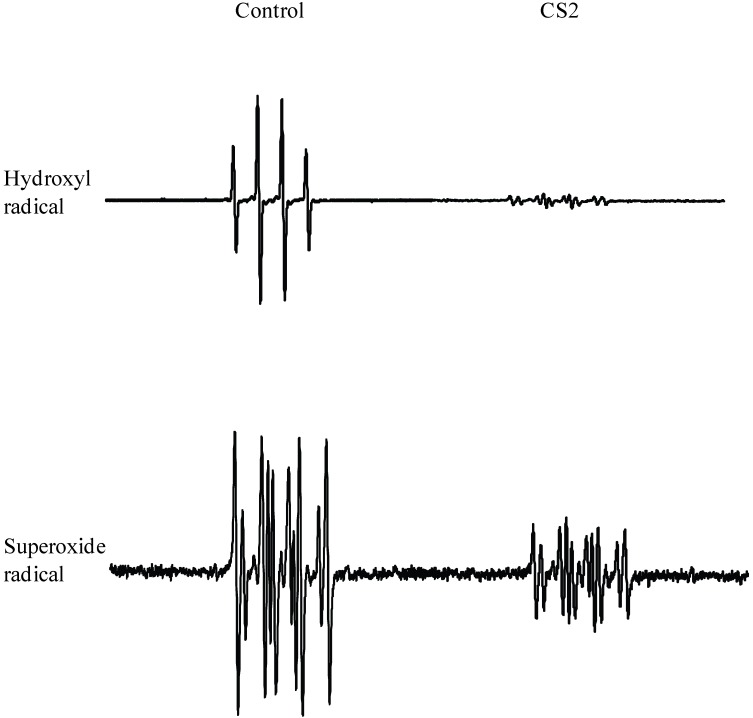
Comparison of ESR spectra of hydroxyl and superoxide radicals following treatment with different LMW chitosans at a concentration of 200 μg/mL.

The potential of LMW chitosans to scavenge hydroxyl and superoxide radicals was monitored by ESR spectroscopy with spin trapping agents. Spectra obtained in the absence of treatment groups verified that the DMPO trapping strategy is functioning properly in the assay methods employed by the resulting clear spin adducts, DMPO/^•^OH and DMPO/ O2^•‾^ radicals. Table 2 summarizes the scavenging effects of the above radical species by LMW chitosans by comparing their ESR signal adducts with those of non-treated controls. In the presence of LMW chitosans, both radicals were scavenged to different degrees in a dose-dependent manner. The antioxidative effect was observed to be in the order: CS2 >> CS1 > CS3 > CS4 (μg/mL). LMW chitosans are able to scavenge ^•^OH more effectively in a dose dependent manner, resulting the least intense DMPO/^•^OH spectra for all concentrations tested. CS2, at a concentration of 100 μg /mL, scavenged ^•^OH and O2^•‾^ radicals to different degrees, as shown in Figure 4. Furthermore, a comparative analysis of the intensities of spectra obtained in the presence of LMW chitosans clearly indicated a significant difference (*p* > 0.05) between the scavenging potency of CS2 and the other LMW chitosans.

**Table 2:** Effect of LMW chitosans on the scavenging of different radical species, as assessed by ESR spectroscopy.

Conc. (g/mL)	Scavenging percentage (%)								
	Hydroxyl radical				Superoxide radical			
	CS1	CS2	CS3	CS4	CS1	CS2	CS3	CS4	
100	53.7 ± 3.5	65.1 ± 4.5*	50.5 ± 3.8	49.5 ± 4.7	39.7 ± 4.8	48.2 ± 5.8*	33.5 ± 5.4	30.8 ± 5.2	
200	80.7 ± 4.5	92.1 ± 4.6*	78.1 ± 4.6	75.1 ± 3.1	65.1 ± 4.6	80.1 ± 5.6*	56.1 ± 5.8	50.1 ± 5.6	

*P < 0.05, compared with other LMW chitosans.

### 3.4. Release study of TPH and scavenging activity of chitosans-TPH tablet

**Figure 5:**
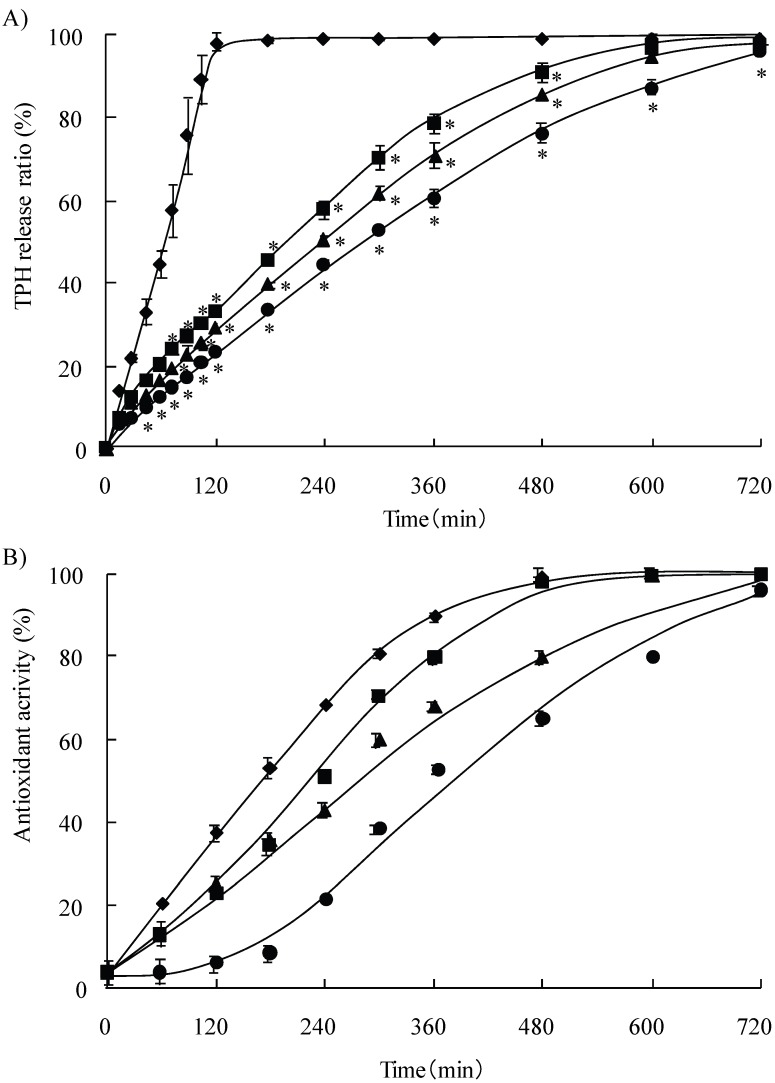
Release profiles of TPH from tablets containing various LMW chitosans in distilled water and antioxidant activity of chitosans TPH tablet. CS1 (♦), CS2 (■), CS3 (▲), and CS4 (●). * *P* < 0.05 compared to CS1 (♦).

Figure 5A shows the dissolution profiles for theophylline (TPH) from matrix tablets containing LMW chitosans in distilled water in the presence of ABTS radicals. 100% of the drug was released from the CS1 tablet (TPH: CS1 = 200 mg : 200 mg) within 2 h. On the other hand, in the case of tablets containing the other LMW chitosans (TPH : CSs = 200 mg : 200 mg), 100% of the drug was released within 12 h. Identical results were obtained when distilled water was used without ABTS radical solutions. As shown Figure 5B, the CS2-TPH tablet showed the highest scavenger activity of the three chitosans tablets (CS2-CS4) in the extended-release of TPH. Given the fact that the CS2 clearly had the highest antioxidant activity (Figure 2,Figure 3,Figure 4), the CS2-TPH tablet could be useful as an extended-release tablet with a high antioxidant activity. 

### 3.5. Discussion

Antioxidants are substances that delay the oxidation process, inhibit chain polymerization reactions that are initiated by free radicals and other subsequent oxidation reactions and thereby aid in preventing cancer, heart disease, diabetes mellitus, neurodegenerative and inflammatory diseases [19,20]. Although synthetic antioxidants appear to hold considerable promise, their toxicity and side effects rule out their extensive clinical use. Therefore, interest has developed in identifying natural and safe antioxidative agents from natural sources. Among the various naturally occurring substances, polysaccharides appear to be useful candidates for non-toxic substances with antioxidant activity [9,21].

Chitosan is a naturally occurring cationic polyelectrolyte with gel-forming properties, but also has antioxidant properties. A number of studies have demonstrated that molecular weight, as well as the degree of deacetylation, are both potent determinants of action of LMW chitosans (MW: 1–10 kDa) in biological systems [22]. In a previous study, we measured the ability of 2.8 kDa low MW chitosan preparations to protect HSA from peroxyl radicals and also showed that the administration of chitosan supplements (MW: 20–40 kDa) to human volunteers prevented HSA oxidation *in vivo* (Anraku *et al.*, 2008; Anraku *et al.*, 2009) [11,14]. However, information on the antioxidant properties of various LMW chitosans (MW: 10–100 kDa) was not available at that time. In addition, if these chitosans have antioxidative effects, they would have potential use in extended-release systems.

In this study, DPPH was used to determine the proton scavenging activity of four different MW chitosans. The dose-response curves for the chitosans examined are shown in Figure 2. The concentration of the chitosans was a factor in their DPPH-scavenging activity. CS2 exhibited the highest radical-scavenging activity, followed by CS1, CS3, and CS4. The effect of antioxidants on DPPH scavenging is thought to be due to their hydrogen-donating abilities. DPPH is a stable free radical and accepts an electron or hydrogen radical to form a stable diamagnetic molecule [23]. In the present study, CS2 showed excellent scavenging activity for DPPH, which can be attributed to its strong hydrogen-donating characteristics. Figure 3 depicts the reducing power of several chitosans based on the TPAC test. The reducing capacity of a compound can serve as a significant indicator of its potential antioxidant activity. The reducing power of CS2 was more pronounced than that of the other chitosans. The order of reducing power for the chitosans tested was CS2 > CS1 > CS3 > CS4; an order similar to their radical scavenging activities. The findings also showed that the LMW chitosans were effective in scavenging both hydroxyl and superoxide radicals (Figure 4). Hydroxyl radicals were generated using the Fenton reaction and were visualized by ESR spectrometry. The intensity of the ESR signal is decreased in the presence of ^.^OH scavengers, which compete with DMPO for ^.^OH. Hydroxyl radicals are highly reactive and can cause extensive biological damage. Of all the LMW chitosans tested, CS2 was found to be an effective scavenger of hydroxyl radicals at relatively low concentrations. Superoxide radicals were generated using a UV irradiated riboflavin/EDTA system. Interestingly, all of the chitosans were more effective in scavenging hydroxyl radicals than superoxide radicals. In a previous study, LMW chitosans (MW; 1-10 kDa) with a relatively higher degree of deacetylation and lower MW were found to have higher radical scavenging activities on DPPH, hydroxyl and superoxide radicals [24,25]. In this study, we initially hypothesized that a LMW chitosan with a MW of 38 kDa would have the highest antioxidant activity of LMW chitosans with MW in the range of 10 to 100 kDa. In general, chitosans show considerable hydrogen bonding on N2–O6 and O3–O5. High MW chitosans have compact structures, thus making the overall effect of their intra-molecular hydrogen bonds stronger. The strong effect of intra-molecular hydrogen bonding decreases the reactivity of hydroxyl and amino groups. On the contrary, low molecular weight chitosan has a more compact structure, thus making the overall effect of intra-molecular hydrogen bonding less effective. In particular, a sutructure of LMW chitosan with a MW of 38 kDa may be the weakest effect of intra-molecular hydrogen bonding. The findings reported herein confirm this and suggest that CS2 would serve to prevent or inhibit oxidative stress.

Various controlled drug delivery systems have been developed, in attempts to improve the efficacy of an administered drug, in terms of decreasing undesirable side effects and increasing patient compliance (Nellore *et al*., 1998; Kranz *et al*., 2005) [26,27]. Among the various polymers that have been considered, several studies have reported on the possible use of chitosan as a tablet excipient. It has been tested for use as a vehicle in sustained release tablets, a direct compressible diluent, a tablet disintegrant and a tablet binder [28,29,30]. In addition, attempts have been made to improve the properties of chitosan property by developing salt derivatives. Spray-dried chitosan microspheres, prepared using acetic acid as a solvent, loaded with insulin for protein delivery and chitosan microspheres, loaded with dexamethasone have been prepared, as well as spray-dried lactose composite particles containing an ion complex derived from an alginate-chitosan mixture [31]. There are several patents describing the preparation and use of chitosan microparticles prepared by spray drying acetic acid solutions of chitosan (MW 2392, 42,000, 142,000 Da) [32,33,34]. Therefore, the use of LMW chitosans in conjunction with drugs might also permit a sustained drug release on a level comparable or superior to that achieved using preformed complexes. As shown in Figure 5A, the rate of drug dissolution in tablets containing more than 30 kDa LMW chitosan was very slow, compared with that for CS1 tablets containing chitosan with a molecular weight of under 30 kDa. Theophylline (TPH) is animportant drug that is used in the treatment of asthma and recent studies indicate that it has anti-inflammatory effects [35,36]. Thus, considerable interest has developed in terms of its new effect in the treatment of asthma. Unfortunately, since the drug has a short half-life (6 h), conventional dosages must be administered 3-4 times per day in order to avoid large fluctuations in plasma concentrations, which led to poor patient compliance [37]. In addition, its therapeutic index is narrow (10–20 μg/mL). The therapeutic effects of TPH require a plasma TPH concentration of at least 5–10 μg/mL and toxic effects are frequently observed at concentrations above 20 μg/mL. Therefore, sustained release dosage forms, prepared using LMW chitosans could potentially overcome these drawbacks. Given the fact that, when LMW chitosans with molecular weights in excess of 30 kDa are used, TPH is released within 12 h, which shows an optimal therapeutic use of TPH (Figure 5A) and that oxidative stress is a central factor in many chronic inflammatory diseases such as severe asthma and chronic obstructive pulmonary disease (COPD), the CS2-TPH tablet might be useful as an extended release matrix tablet which also has a high antioxidant activity. In fact, the CS2-TPH tablet showed the highest scavenge activity of the three chitosans tablets (CS2-CS4-TPH) for the extended-release of TPH (Figure 5B).

## 4. Conclusions

Based on the findings presented herein, it can be concluded CS2 (MW 38 kDa) is a very effective inhibitor for oxidative stress in various LMW chitosans (MW: 10-100kDa). The findings also suggest that a CS2-TPH tablet shows promise for use, not only as an extended release tablet with a table with high antioxidant activity that is also safe and non-toxic.
